# Chromium removal from tannery effluents by adsorption process via activated carbon chat stems (*Catha edulis*) using response surface methodology

**DOI:** 10.1186/s13104-021-05855-7

**Published:** 2021-11-25

**Authors:** Yigezu Mekonnen Bayisa, Tafere Aga Bullo, Desalegn Abdissa Akuma

**Affiliations:** School of Chemical Engineering, Jimma Institute of Technology, Jimma University, Jimma Oromia, Ethiopia

**Keywords:** Adsorption of chromium, Chat stems activated carbon, Response Surface Methodology, Tannery wastewater treatment

## Abstract

**Objective:**

In tannery processing, water consumption is high, which generates wastewater as a by-product and numerous pollutants such as chromium heavy metals that make adverse effects of water bodies and the surrounding environment. This study analyzed, chromium (VI) removal from wastewater through activated carbon chat stem was investigated. Adsorption is a common treatment method via activated carbon due to its cost-effective, profitable, and removal efficiency of these heavy metals.

**Results:**

The proximate analysis of moisture content of chat stem has 6%, activated carbon ash content of 17.35%, volatile materials of 20.12%, and fixed carbon contents of 56.53%, which are well-matched the standards quality of activated carbon. As the process parameter varies, the increment of the chromium removal efficiency was from 62.5 to 97.03%. The maximum adsorption efficiency was observed at 30 g/L dosage of the adsorbent, at pH 4, and contact time at 180 min of activated carbon from chat stem waste was found 97.03%. FTIR was used to characterize the surface of the chat stem before and after adsorption. Langmuir and Freundlich are used for short contact time’s adsorption isotherm 0.9839 and 0.9995 respectively, which conformed, no visible change in the corrosion state.

**Supplementary Information:**

The online version contains supplementary material available at 10.1186/s13104-021-05855-7.

## Introduction

Nowadays, water pollution is increasing rapidly, due to urbanization, population growth, and quick industrialization, at an increasing degree, frequently discharge a huge amount of potentially toxic chromium metals and untreated waste into surface and groundwater [1–4]. The waste released from tanneries process consumed 60–70% of total chromium, whereas, the rest of 30–40% remains unconsumed. This unconsumed chromium goes away with the industrial pollution due to the tanning process with Cr-salt and consequently, large quantities of Cr are being discharged by polluting surface waters, soil, air, land, and groundwater [5–8]. This, adsorption is the most common technique for the removal of chromium from wastewater by preparing activated carbon from chat stems found in jimma city, Ethiopia. Activated carbons are more effective in the removal of heavy metals due to their non-biodegradability and persistence in the environment for the removal of both organic and inorganic contaminants from wastewater [9–12].

In this study, we investigate using valuable activated carbons chat stem to treat wastewater generated from tannery effluents, for the removal of harmful chromium by using low-cost adsorbent materials under different parameters.

## Main text

### Methods

**Fig. 1:**
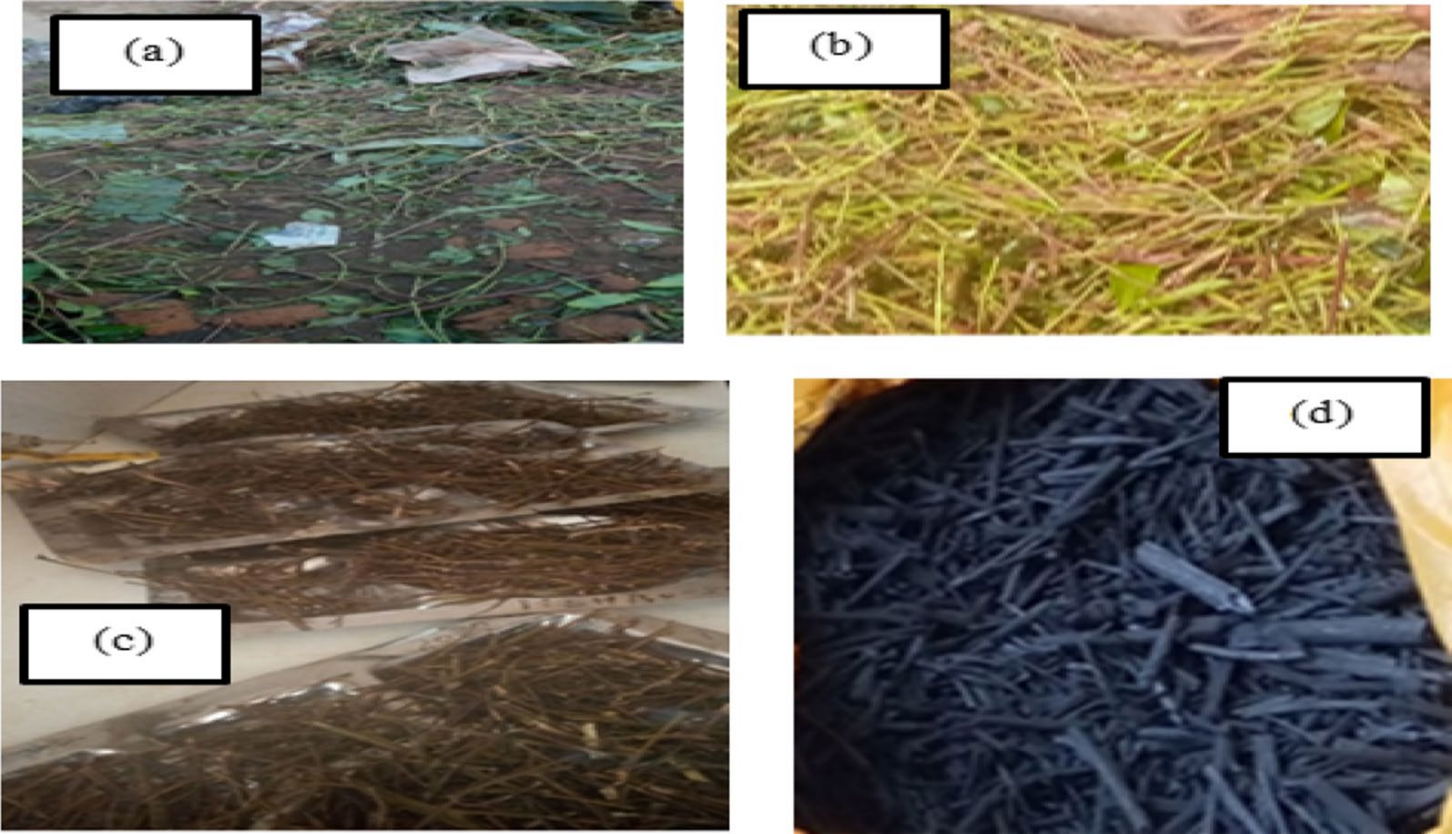
**a** Chat stem sample collection, **b** after washing, **c** drying in an oven, **d** after pyrolysis

The dried powder of chat stem was carbonized in a furnace at temperature of 600 $^\circ{\rm C}$. Then, the samples was activated by 0.5 M HCl by soaking with 98% of concentrated H_2_SO_4_ for 24 h. Then, 1% of dilute HNO_3_ was washed the prepared activated carbon and neutralized using diluted water. Finally activated carbon kept for the adsorption experiment (Fig. 1).

The adsorption experiment was performed by adding activated carbon of chat stem ranged from 10 to 30 g/L to a standard solution of Cr (VI) which was prepared by dissolving an appropriate amount of K_2_Cr_2_O_7_ in distilled water. This solution was diluted to obtain a 200 mg/L of Cr (VI) concentration,, pH adjusted using dilute 0.5 M HCl and agitated at 500 rpm placed in the flasks and shakes for 3 h at 30 ºC [13]. For effect of pH on adsorption capacity, the adsorptions were tested at pH ranged from 1.0 to 7.0 using a 200 mg/L Cr (VI) concentration. Adsorption isotherms were carried out with different initial concentrations of Cr (VI). The percentage removal of Cr (VI) by adsorption calculated using equation:

1 $$Adsorption \, (\% ) \, = \frac{CI - CF}{{CI}} \times 100$$

where; C_I_ and C_F_ are the initial and final chromium concentrations.

### Adsorbent characterization

#### Moisture content

2 g weighed samples were added to the crucible and weighed. The samples were kept in the oven at 105 °C for 12 h and kept in the desiccators. The weights sample recorded before and after drying and burnt the samples into oven and furnace respectively. Moisture content calculated as;

2 $$Moisture \, content \, \left( \% \right) = \frac{M0 - M}{{M0}} \times 100$$

where; M0 = mass of crucible plus sample; M = mass of crucible plus dried sample.

#### Ash content

A sample of 2 g weighed and kept in a muffle furnace for one-half hours at a temperature of 650 °C, and occupied out and kept in desiccators for half-hour to cool. Again, measure the weight samples and calculate ash content by Eq. (2).

#### Volatile matter

Using the same samples in a furnace at a temperature of 800 °C for 10 min, and samples were taken out and kept in the desiccators for half-hour to cool. Volatile matter calculated as;

3 $$V = \frac{{100\left( {B - F} \right) - M\left( {B - G} \right)}}{{\left( {B - G} \right)\left( {100 - M} \right)}} \times 100$$

where, V = volatile matter (%), B = mass of crucible plus sample, F = mass of crucible plus dried sample, G = mass of empty crucible, M = moisture content of the samples.

#### Fixed carbon

Calculated using;

4 $$\% \, Carbon \, = 100 \, {-} \, \left( {\% \, moisture \, content \, + \, \% \, volatile \, content{ + }\% \, ash \, content} \right).$$

#### Isotherms of adsorption

Adsorption isotherms define mathematical models of the dispersal of adsorbate species among fluid and solid phases [14]. To construct adsorption isotherm of chat stem, an experiment was carried out by varying the initial concentration of metal ions as 8, 14, and 18 mg/L in 2.2 g of dose with 100 ml solution of the metal ions. Langmuir isotherm is described by equation [15]:

5 $$qe = \frac{qmkace}{{1 + kace}},$$

where; ${q}_{e}$ amount removal to adsorbent (mg/g) at concentration equilibrium of ${C}_{e}$ (mg/L), ${q}_{m}$ maximum adsorption for monolayer complete capacity (mg/g), Ka constant adsorption equilibrium (L/mg). The linearized Langmuir isotherm allows the adsorption calculation by equation [15];

6 $$\frac{ce}{{qe}} = \frac{1}{{\left( {qmka} \right)}} + \frac{ce}{{qm}}.$$

The necessary features of Langmuir isotherm equation can be explained in terms of a constant dimensionless equilibrium factor (K_L_) as;

7 $$kL = \frac{1}{1 + kac0},$$

where, C_o_, K_a_ and K_L_, is the initial adsorbate concentration, Langmuir constant (L/mg), and adsorption process nature respectively. Freundlich isotherm is described by equation [12];

8 $$qe = KFC_{e}^{\frac{1}{n}},$$

where; n and ${K}_{F}$ incorporating constants parameters that affect the adsorption intensity and capacity respectively, and calculated plot slope and intercepts. The isotherm Freundlich linear function, represented as:

9 $$log\left( {qe} \right) = \frac{1}{n}log\left( {Ce} \right) + log\left( {KF} \right).$$

#### Analysis of variance (ANOVA)

The Design-Expert software version 11, using RSM was used to determine the influences of operating variables for adsorption and maximum percentage removal efficiency of chromium from tannery wastewater. A BBD with three numerical factors on three levels was used. They consisted of 17 randomized runs. In this study, the effects of dosage of adsorbent varying from 10 to 30 g/L, contact time from 30 to 180 min, and effects of pH from 1 to 7 were investigated.

### Results and discussion

#### Proximate analyses of Chat stem activated carbon

Moisture content for activated carbon was 6%. In this regard, the low moisture content of the activated carbon was preferable when related to that of commercial activated carbon [10]. Ash content for activated carbon was 17.35%. A high quality of ash values of activated carbon indicates that lower capacity and efficiency of the adsorption, whereas, low ash content is the critical concern for wastewater purification through adsorption. Volatile matter for activated carbon was 20.12%. Fixed carbon for activated carbon was 56.53%. The prepared activated carbon from chat stem contained more carbonous material and indicates a good quality sign for adsorbent [1]. Additional file 1: Table S1 summarized proximate analyses of chat stem activated carbon for the adsorbent.

#### ANOVA analysis

Based on the ANOVA analysis the parameter that significantly affected the efficiency of chromium removal from wastewater was shown in Additional file 2: Table S2. The F-value of 178.50 implies the model is significant; P-values less than 0.0500 show the model terms and all process parameters and interaction effects are significant and Lack of F-value of 1.90 implies Lack of Fit is not significant.

#### Factors affecting the parameters of adsorption

**Fig. 2:**
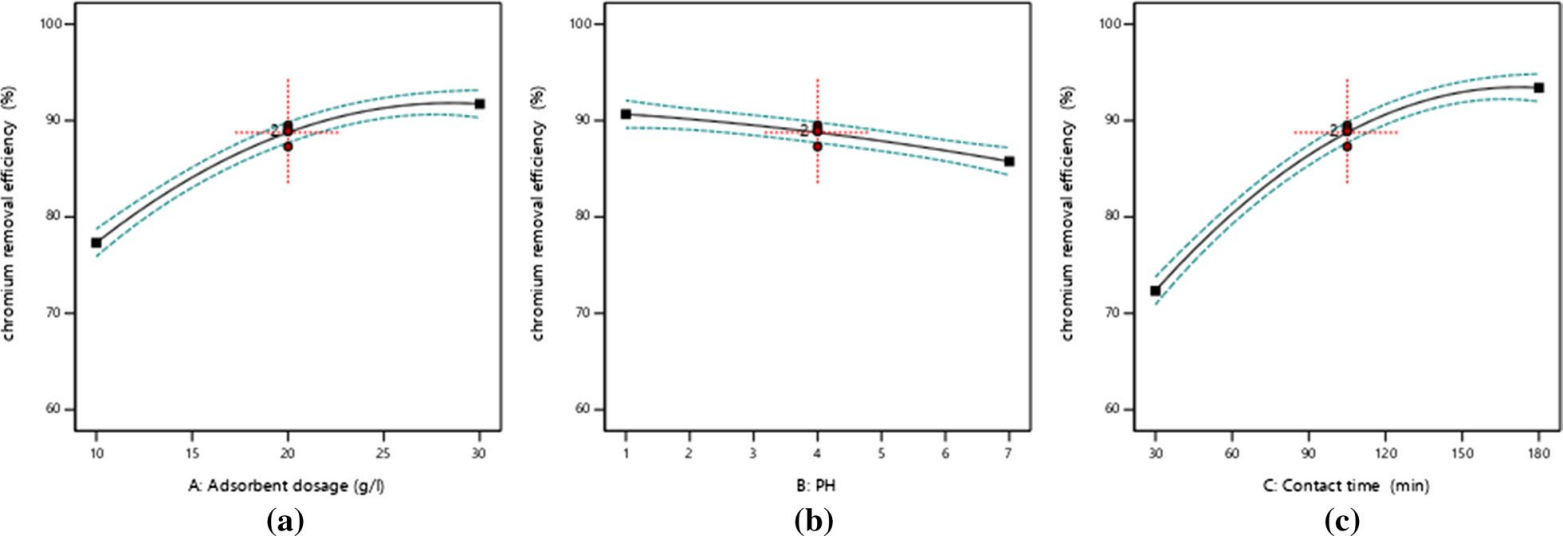
**a** Effect of adsorbent dose, **b** Effect of pH, and **c** Effect of contact time on Cr removal

Effect of dose of the adsorption primarily associated with the surface of the adsorbent which is interrelated to the amounts of the adsorbent and adsorption capacity increases with an increase in surface of activated carbon [10, 11]. The results show in Fig. 2a as the dosage of activated carbon chat stem increase the adsorption efficiency was decreased due to increased surface area of the adsorbent, and the adsorption of contact sites also increases. In other way, at high adsorbent concentration, the amount of adsorbate metal ion per unit mass of the adsorbent decreases with increase in the adsorbent dose and this may be due to the available metal ion to be adsorbed is not adequate to entirely cover the accessible active sites of the adsorbents that leads to the reduction of metal ion uptake. The maximum removal of chromium was observed at 30 g/L of activated chat stem dose, 180 min of contact time, and at pH of 4.

The pH measures the degree of ionization of a species affected adsorption by pH [10, 16]. The result in Fig. 2 (b) shown that optimal removal of Cr^6+^ was attained at pH 1.0 for 180 min contact time. The adsorption efficiency increases with a decrease in pH of acid, due to excess hydrogen ions is neutralized of negative charges on the surface of the adsorbent by forming the diffusion of hydrogen chromate ion (HCr_2_O^7*−*^) in the solution at lower pH for a higher abundant anionic form of Cr^6+^.

The contact time investigations needed to reach the equilibrium point dependent on the initial and final concentrations of solution at different times, which was found to increase rapidly in the initial stage [10, 11]. In this study that increasing contact time leads increases the adsorption efficiency but removal and adsorption capacity was not changed as contact time increase beyond the design point. Figure 2c, the percentage removal of chromium at a contact time of 50 min was 70.7% and it was increased with time until equilibrium was achieved but at the dosage of activated carbon chat stem of the 20 g/L as time increases to 180 min 97.8% of chromium removal efficiency was achieved.

#### Interaction’s effect

The response surface curves representing the interaction effects of two variables, i.e. adsorbent dosage with pH and pH with contact time on adsorption of chromium were plotted as shown in Additional file 5: Figure S1(a) shows that maximum adsorption of chromium was attained at a high adsorbent dosage (30 g/L), and considerably low pH. Additional file 5: Figure S1(b) shows maximum adsorption of chromium at relatively low pH and high contact time. The adsorption equilibrium of chromium compounds was obtained, after 105 min contact time and 25 g adsorbent dose with adsorption of approximately 96% of chromium compounds. In general, at sufficient contact time the increment in adsorbent dose from 10 to 30 g/L results in an increment of chromium removal efficiency from 62.5% to 97.03%, whereas the adsorption capacity decreased from 4.26 to 1.55 mg/l. The compared adsorption capacities of Chat Stem to remove Cr (VI) with other adsorbents are shown in Additional file 3: Table S3. Hence, Chat Stem indicated better removal efficiency than other adsorption.

#### FTIR analysis

**Fig. 3:**
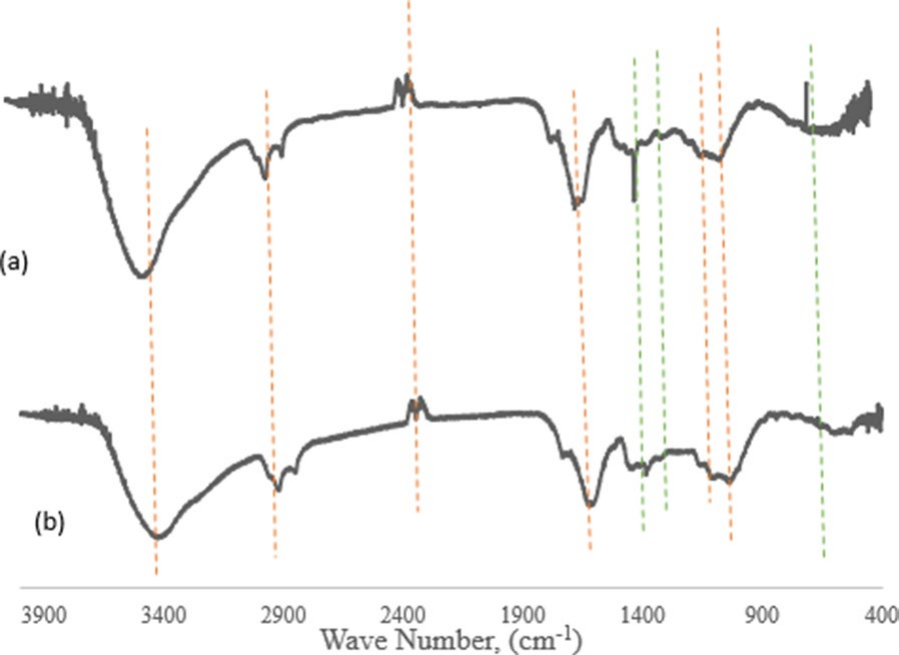
FTIR graph of the raw chat stem **a** before and **b** after adsorption

Functional groups of the chat stem before and after adsorption were identified using Fourier transforms infrared (FTIR) transmission as shown in Figs. 3a and b. Broadband noticed from 3650 to 3120 cm^−1^ corresponds to the hydroxyl functional group, the bands at 2920 cm^−1^ assigned to asymmetric C–H stretching, region between 1700 cm^−1^ and 1490 cm^−1^ were attributed to C=C symmetrical stretching of pyrone groups, and the band observed at 1632 cm^−1^ was assigned to carbonyl C–O present in carbonyls [12]. Figure 3b shows that many functional groups shifted to different frequency levels or disappeared after adsorption, indicating the possible involvement of those groups for uptake of the adsorbate. It can be observed that the sharp and intense peak at around 3,434 cm^−1^ was shifted to a lower frequency level of 3423 cm^−1^ and after adsorption, it was broad, which represented that the hydrogen-bonded -OH group was involved for binding adsorbate from wastewater.

#### Method validation

The method validation is essential to determine the developed methodology is fully aligned with the objectives that have been set and are considered one of the main quality control tools. The parameters used in the validation process are selectivity, linearity, and accuracy. The selectivity of the method by mid-infrared spectroscopy was demonstrated by the presence of a vibratory band, and the absence of analytical signals in this region of the absorption spectrum of the region, as can be seen in the overlap of the spectra of chat stem before and after adsorption were identified (Fig. 3).

#### Isotherm adsorption

The plotted graph shows the relationship between Ce and Ce/qe, and log Ce and log qe in X and Y-axis using Langmuir and Freundlich equation respectively. Additional file 4: Table S4 shows Cr (VI) adsorption isotherm ions. The result shown in Additional file 6: Figure S2, using Eq. 6, the q_m_ and K_a_ observed constants are 2.083 mg/g and 0.0925, respectively. The value of K_L_ (0 < 1/ (1 + 0.0925C_o_) < 1). This means that chat stem activated carbon for removal of Cr (VI) ions from wastewater is a promising adsorbent. The model equation of Freundlich fits with experimental data was examined, from graph of log q_e_ Vs log C_e_, to generate the intercept value of log K_F_ and the slope of 1/n. Additional file 6: Figure S2, and Eq. 9, the Freundlich constants K_F_ and n are 0.2218 and 1.503, respectively. The value of 1/n is 0.6653, this value between 0 and 1 and n > 1, which shows the chat stem, can adsorb Cr (VI) ions effectively. Langmuir and Freundlich correlation constants (R^2^) are 0.9839 and 0.9995 respectively, therefore adsorption isotherms can fitted the chromium adsorption for chat stem.

### Conclusion

Chromium is a heavy metal, which is very harmful to human health, animals, and environmental stability of the surrounding affected extremely when its absorption standard level goes beyond the permissible level. Chat stem activated carbon used as an adsorbent to remove chromium from wastewater since adsorption is a real treatment method due to; environmentally friendly, economical, and highly effective adsorbent. The proximate analysis of moisture content, ash content, volatile materials, and fixed carbon contents has 6%, 17.35%, 20.12%, and 56.53% respectively. The maximum removal efficiency of chromium by adsorption was observed at 30 g/L dosage of the adsorbent, at pH 4, and contact time at 180 min was found 97.03%. Investigational data on the chromium adsorption by Langmuir and Freundlich adsorption isotherm were acceptable since the correlation coefficients (R^2^) were 0.9839 and 0.9995 respectively.

## Limitations

The limitations of this study that need to be addressed are influence of particle size and SEM was studying before and after adsorption with Chromium.

## Supplementary Information

**Additional file 1: Table S1**. Summarized proximate analyses of chat stem activated carbon used for adsorbent.

**Additional file 2: Table S2.** ANOVA analysis for process operating parameter of Cr (VI) removal.

**Additional file 3: Table S3.** Comparison of the adsorption removal efficiency of Cr (VI) from wastewater in Chat Stems activated carbon with other adsorbents.

**Additional file 4: Table S4**. Adsorption isotherm of Cr (VI) ions.

**Additional file 5: Figure S1.** Interaction effects between (a) adsorbent dosage and pH; (b) pH and contact time interaction’s effect result for chat stem.

**Additional file 6: Figure S2.** Langmuir adsorption isotherms and Freundlich adsorption isotherm for Cr (VI) ions

## Data Availability

All data generated or analyzed during this study are included in this article [and its supplementary information files].

## Abbreviations

ANOVA: Analysis of variance
BBD: Box-Behnken design
Cr: Chromium
FTIR: Fourier transforms infrared
SEM: Scanning electron microscopy
RSM: Response Surface Methodology
