# Supplementary material for: Chromium removal from tannery effluents by adsorption process via activated carbon chat stems (Catha edulis) using response surface methodology
Source: BMC Res Notes. 2021 Nov 25;14:431. doi: 10.1186/s13104-021-05855-7 (PMC8620636; doi:10.1186/s13104-021-05855-7)
Supplement: Supplementary file 1 — Additional file 1: Table S1. Summarized proximate analyses of chat stem activated carbon used for adsorbent. [file 13104_2021_5855_MOESM1_ESM.docx]

Table S1 Summarized Proximate analyses of chat stem activated carbon used for adsorbent

| **Proximate analysis contents** | **Mass in %** |
| --- | --- |
| Moisture | 6.0 |
| Volatile matter | 20.12 |
| Ash content | 17.35 |
| Fixed carbon | 56.53 |
