# Supplementary material for: Chromium removal from tannery effluents by adsorption process via activated carbon chat stems (Catha edulis) using response surface methodology
Source: BMC Res Notes. 2021 Nov 25;14:431. doi: 10.1186/s13104-021-05855-7 (PMC8620636; doi:10.1186/s13104-021-05855-7)
Supplement: Supplementary file 2 — Additional file 2: Table S2. ANOVA analysis for process operating parameter of Cr (VI) removal. [file 13104_2021_5855_MOESM2_ESM.docx]

**Table S2. ANOVA analysis for process parameter of Cr (VI) removal**

| **Source** | Sum of square | df | Mean square | St. deviations | F- value | P-value |  |
| --- | --- | --- | --- | --- | --- | --- | --- |
| **Model** | 1618.80 | 9 | 179.87 |  | 178.50 | < 0.0001 | significant |
| A-dosage | 415.15 | 1 | 415.15 | 0.3536$\pm$ 0.035 | 411.99 | < 0.0001 |  |
| B-PH | 48.02 | 1 | 48.02 | 0.3536$\pm$ 0.056 | 47.65 | 0.0002 |  |
| C-Contact time | 886.84 | 1 | 886.84 | 0.3536$\pm$ 0.026 | 880.08 | < 0.0001 |  |
| AB | 18.06 | 1 | 18.06 | 0.5000 $\pm$ 0.012 | 17.92 | 0.0039 |  |
| AC | 5.13 | 1 | 5.13 | 0.5000 $\pm$ 0.083 | 5.09 | 0.0586 |  |
| BC | 7.02 | 1 | 7.02 | 0.5000 $\pm$ 0.048 | 6.97 | 0.0334 |  |
| **Residual** | 7.05 | 7 | 1.01 |  |  |  |  |
| Lack of Fit | 4.14 | 3 | 1.38 |  | 1.90 | 0.2715 | not significant |
