# Supplementary material for: Chromium removal from tannery effluents by adsorption process via activated carbon chat stems (Catha edulis) using response surface methodology
Source: BMC Res Notes. 2021 Nov 25;14:431. doi: 10.1186/s13104-021-05855-7 (PMC8620636; doi:10.1186/s13104-021-05855-7)
Supplement: Supplementary file 3 — Additional file 3: Table S3. Comparison of the adsorption removal efficiency of Cr (VI) from wastewater in Chat Stems activated carbon with other adsorbents. [file 13104_2021_5855_MOESM3_ESM.docx]

Table S3. Comparison of the adsorption removal efficiency of Cr (VI) from wastewater in Chat Stems Activated Carbon with other adsorbents.

| **No.** | **(Adsorbents )** | **pH** | **Adsorbent dosage** | **Contact time** | **Removal efficiency** | **References** |
| --- | --- | --- | --- | --- | --- | --- |
|  | Acid treated lignite coal | 1 | 3.5 g | 4 hr | 49.94% | [17] |
|  | Acid treated lignite coal | 1 | 3.01g | 4 hr | 96.5% |  |
|  | Coffee husk | 2 | 2.4 g/L | 60 min | 83 % | [18] |
|  | Vesicular basalt | 2 | 50 g/L | 3 hr | 81.2 % | [19] |
|  | Chat stem | 4 | 30 g/L | 3 hr | 97.03%. | This study |
