# Supplementary material for: Chromium removal from tannery effluents by adsorption process via activated carbon chat stems (Catha edulis) using response surface methodology
Source: BMC Res Notes. 2021 Nov 25;14:431. doi: 10.1186/s13104-021-05855-7 (PMC8620636; doi:10.1186/s13104-021-05855-7)
Supplement: Supplementary file 4 — Additional file 4: Table S4. Adsorption isotherm of Cr (VI) ions. [file 13104_2021_5855_MOESM4_ESM.docx]

**Table S4.** Adsorption isotherm of Cr (VI) ions

| **Initial concentration (** **(mg/L))** | **Final conc.**   **(mg/L)** | **Adsorption capacity (****) (mg/g)** |  | **Log**  | **Log**  |
| --- | --- | --- | --- | --- | --- |
| 7 | 4.24 | 0.45 | 6.7 | 0.5 | -0.3 |
| 13 | 5.532 | 0.76 | 8.0 | 0.85 | -0.1 |
| 17 | 7.659 | 0.95 | 9.5 | 0.95 | -0.03 |
