# Supplementary material for: Chromium removal from tannery effluents by adsorption process via activated carbon chat stems (Catha edulis) using response surface methodology
Source: BMC Res Notes. 2021 Nov 25;14:431. doi: 10.1186/s13104-021-05855-7 (PMC8620636; doi:10.1186/s13104-021-05855-7)
Supplement: Supplementary file 5 — Additional file 5: Figure S1. Interaction effects between (a) adsorbent dosage and pH; (b) pH and contact time interaction’s effect result for chat stem. [file 13104_2021_5855_MOESM5_ESM.docx]

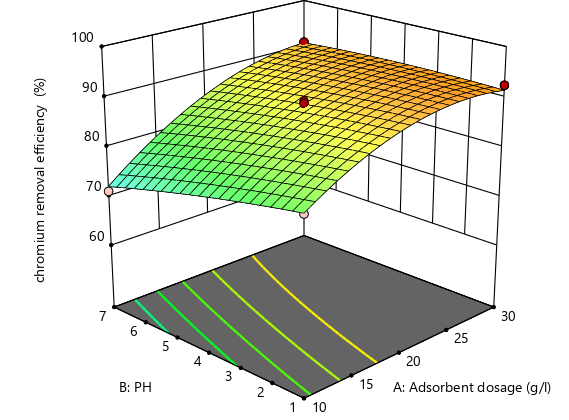

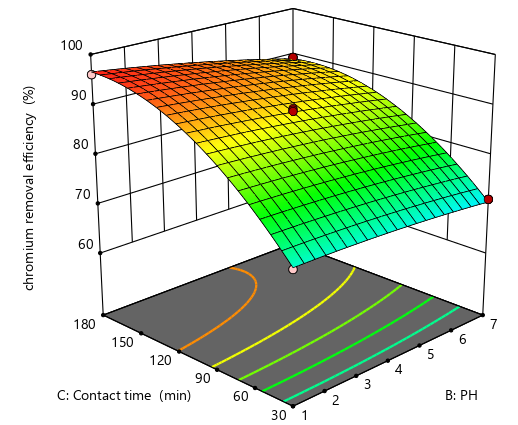


(a)

(b)

Figure S1 Interaction effects between (a) adsorbent dosage and pH; (b) pH and contact time interaction’s effect result for chat stem.
