# Supplementary figures and images for: Chromium removal from tannery effluents by adsorption process via activated carbon chat stems (Catha edulis) using response surface methodology
Source: BMC Res Notes. 2021 Nov 25;14:431. doi: 10.1186/s13104-021-05855-7 (PMC8620636; doi:10.1186/s13104-021-05855-7)

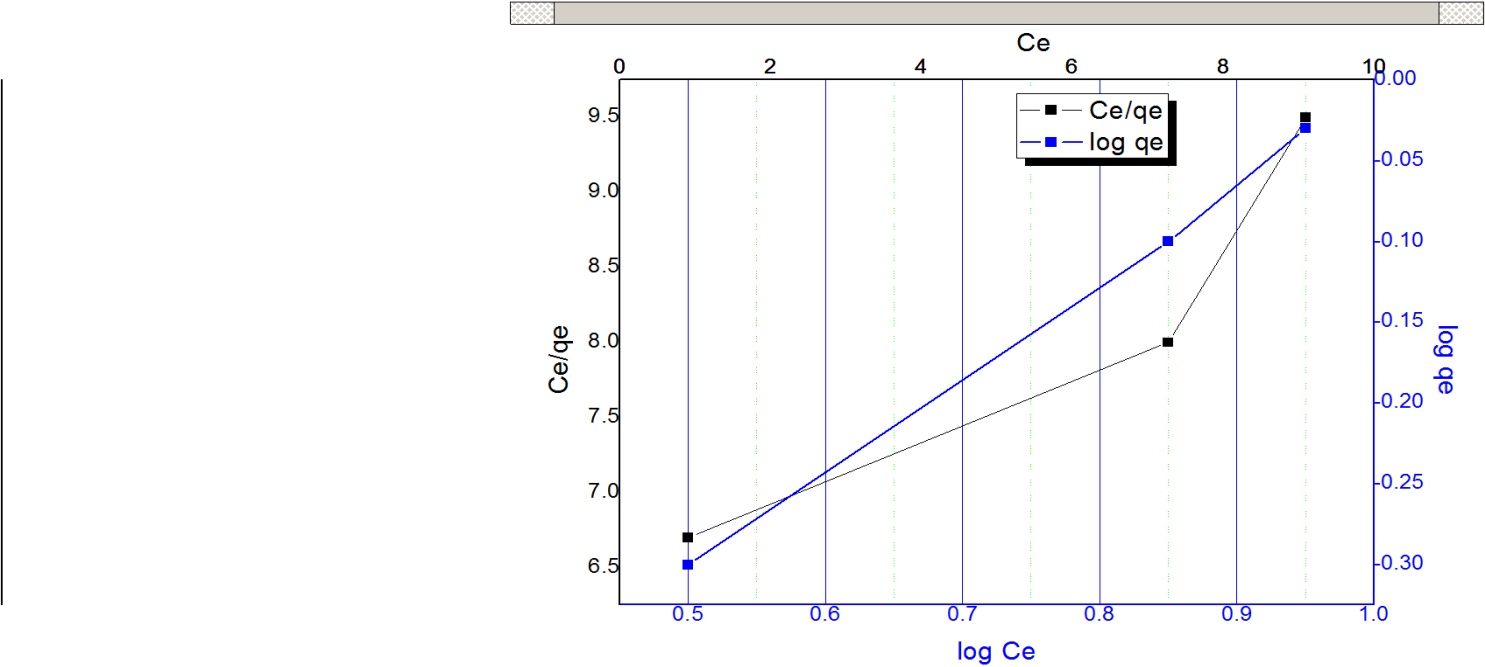


**Figure S2** Langmuir adsorption isotherms and Freundlich adsorption isotherm for Cr (VI) ions

Supplement: Supplementary file 6 — Additional file 6: Figure S2. Langmuir adsorption isotherms and Freundlich adsorption isotherm for Cr (VI) ions [file 13104_2021_5855_MOESM6_ESM.docx]
